# Lithium and Potassium Cations Affect the Performance of Maleamate-Based Organic Anode Materials for Potassium- and Lithium-Ion Batteries

**DOI:** 10.3390/nano11113120

**Published:** 2021-11-19

**Authors:** Kefyalew Wagari Guji, Wen-Chen Chien, Fu-Ming Wang, Alagar Ramar, Endazenaw Bizuneh Chemere, Lester Tiong, Laurien Merinda

**Affiliations:** 1 Graduate Institute of Applied Science and Technology, National Taiwan University of Science and Technology, Taipei 106, Taiwan; gujikefyalew@gmail.com (K.W.G.); chemramar@gmail.com (A.R.); endazenaw27@gmail.com (E.B.C.); ltpw93@hotmail.com (L.T.); merinda795@gmail.com (L.M.); 2 Battery Research Center of Green Energy, Ming Chi University of Technology, New Taipei City 243, Taiwan; 3 Department of Chemical Engineering, Ming Chi University of Technology, New Taipei City 243, Taiwan; 4 Sustainable Energy Center, National Taiwan University of Science and Technology, Taipei 106, Taiwan; 5 Department of Chemical Engineering, Chung Yuan Christian University, Taoyuan 320314, Taiwan; 6 R&D Center for Membrane Technology, Chung Yuan Christian University, Taoyuan 32023, Taiwan

**Keywords:** organic electrode, potassium-ion battery, lithium-ion battery, K-MA anode material

## Abstract

In this study we prepared potassium-ion batteries (KIBs) displaying high output voltage and, in turn, a high energy density, as replacements for lithium-ion batteries (LIBs). Organic electrode materials featuring void spaces and flexible structures can facilitate the mobility of K^+^ to enhance the performance of KIBs. We synthesized potassium maleamate (K-MA) from maleamic acid (MA) and applied as an anode material for KIBs and LIBs, with 1 M potassium bis(fluorosulfonyl)imide (KFSI) and 1 M lithium bis(fluorosulfonyl)imide (LiFSI) in a mixture of ethylene carbonate and ethyl methyl carbonate (1:2, *v*/*v*) as respective electrolytes. The K-MA_KFSI anode underwent charging/discharging with carbonyl groups at low voltage, due to the K···O bond interaction weaker than Li···O. The K-MA_KFSI and K-MA_LiFSI anode materials delivered a capacity of 172 and 485 mA h g^−1^ after 200 cycles at 0.1C rate, respectively. K-MA was capable of accepting one K^+^ in KIB, whereas it could accept two Li^+^ in a LIB. The superior recoveries performance of K-MA_LiFSI, K-MA_KFSI, and Super P_KFSI at rate of 0.1C were 320, 201, and 105 mA h g^−1^, respectively. This implies the larger size of K^+^ can reversibly cycling at high rate.

## 1. Introduction

The favorable electrochemistry of organic materials containing alkali metal ions (namely Li^+^, Na^+^, and K^+^) has led them to serve as energy storage materials in alkali metal ion rechargeable batteries. Certain redox active organic compounds which actively involved in the electrochemistry of a batteries were due to their high source availability, viability of preparation at low temperature, tunable redox behavior upon functional group conversion, low cost, and ready reactions at low potentials, especially when combined with relatively large K^+^ ions [1]. For many purposes, the most convenient way of storing energy is establishment of green energy technologies. For instance, lithium ion batteries (LIBs) are one of the well-known and preferable energy storage systems, due to its long cycle life, high energy density and power density, quite stable cyclic performance which applicable for different electric vehicles, medical, and portable electronic devices. Nevertheless, the low abundance of Li in earth crust (0.0017 wt.%) has led to a search for alternative elements (e.g., Na, Mg, Al, Zn) for use in secondary batteries providing high energy densities [2]. Potassium, in addition to being highly abundant (1.5 wt.%) [3], has recently gained attention for the construction of potassium-ion batteries (KIBs) because it is inexpensive, [4] because the redox potential of the K^+^/K couple (−2.93 V vs. SHE) is comparable with that of Li^+^/Li (−3.04 V vs. SHE), and because its electrode potential is more negative than that of Na^+^/Na (−2.71 V vs. SHE) [5], leading to a high output voltage. Nevertheless, the non-commerciality and high reactivity of K metal makes it challenging to fabricate KIBs. The larger atomic radius of the K^+^ ion (1.38 Å) [6] relative to that of the Li^+^ ion (0.76 Å) [6] means that the former has the lower solvation energy; a relatively weak solvation effect in aprotic solvents [7,8] renders ions with enhanced diffusion kinetics [9,10]. In addition to diffusivity, the conductivity of a K^+^ ion is three times greater than that of a Li^+^ ion in an electrolyte solution [11]. Komaba et al. reported that the standard electrode potential of K^+^/K is −0.15 V lower than that of Li^+^/Li in the intercalation/de-intercalation of K^+^ ions into graphite [12]. Due to the redox potential of K^+^/K being lower than that of Li^+^/Li, KIBs have the ability to operate at voltages higher than those of LIBs and provide higher power densities [13]. Furthermore, K has one more advantage over Li: dendrite-free electrochemistry.

Several organic electrode materials (OEMs) containing carbonyl groups have been investigated for use in LIBs, KIBs, and sodium-ion batteries (NIBs). For instance, terephthalate anions have been employed in LIBs [14,15] and NIBs [14,16]; because they undergo similar charge/discharge mechanisms, potassium terephthalate (K_2_TP), potassium 2,5-yridinedicarboxylate (K_2_PC) [17], 3,4,9,10-perylenetetracarboxylic acid dianhydride (K_2_PTCDA) [18], potassium naphthalene-2,6-dicarboxylate (K_2_NDC) [19], and azobenzene-4,4′-dicarboxylic acid potassium salt (ADAPTS) have also been used in KIBs [20]. When used as a negative electrode, K_2_NDC provided a reversible capacity of 139 mA h g^−1^ at 100 mA g^−1^, measured at a low reduction potential of 0.55 V, with 93% retention of capacity over 300 cycles [19]. Almost the same reversible capacity (134 mA h g^−1^) was generated when using ADAPTS as the anode material of a KIB operated at 0.1C for 100 cycles [20]. In their initial cycling, K_2_TP (at the 0.5/0.7 V redox pair) and K_2_PC (at the 0.6/0.8 V redox pair) provided high reversible capacities of 270 and 245 mA h g^−1^, respectively, with coulombic efficiencies of 46 and 45%, respectively; after 100 cycles, however, these capacities decreased to 158 and 176 mA h g^−1^, respectively [17]. The potassium perylene-3,4,9,10-tetracarboxylate (K_4_PTC) and its composite with carbon nanotubes (K_4_PTC@CNT) have been reported as anode materials for KIBs; of the four C=O groups in K_4_PTC, only two of them accepted K^+^ ions, delivering a capacity of 74 mA h g^−1^ at 50 mA g^−1^ over 300 cycles; in contrast, the K_4_PTC@CNT composite provided a capacity of 97 mA g^−1^ after 500 cycles at a current density of 50 mA g^−1^ [21]. Wang et al. reported a higher storage capacity for K_4_PTC as an anode material: 100 mA h g^−1^ after 500 cycles under a current density of 50 mA g^−1^ [21]. The organic azo compound ADAPTS has been introduced as an anode for KIBs displaying reversibility at low potential, providing a capacity of 109 mA h g^−1^ at a current density of 0.1C over 100 cycles [20]. Zhang studied that anode graphite of LIBs achieved a specific capacity of 365 mA h g^−1^ within 0.0025–0.205 V voltage range at 0.1C rate [22].

Poor rate capability and limited capacity are the major challenges when using OEMs in KIBs, presumably because of the effect of the size of the K^+^ ion. For example, the larger ionic size of K^+^ (1.38 Å) relative to Li^+^ (0.76 Å) results in the K–O bond being weaker than the Li–O bond [23]. Furthermore, the working voltage can decrease because of weaker K···O interactions in organic anode materials [24]. Many studies have found that the insertion potential of the K^+^ ion is lower than those of Li^+^ and Na^+^ ions. For example, the terephthalate salt anode undergoes intercalation/de-intercalation at a voltage of 0.8 V (vs. Li^+^/Li) [25]. Banerjee et al. also found that a metal-organic framework of Li_2_(2,6-NDC) also underwent discharge at a voltage of 0.8 V (vs. Li^+^/Li) [26]. In contrast, the redox potential of a K_2_NDC anode was approximately 0.55 V (vs. K^+^/K) [19]. The potassiation/de-potassiation voltages of several other anode materials for KIBs have also been reported: K_2_PTCDA (ca. 0.64 V) [18], K_2_TP (ca. 0.6 V) and K_2_PC (0.6 V) [19], and potassium 1,1′-biphenyl-4,4′-dicarboxylate (K_2_BPDC) (ca. 0.5 V) [27]. These values indicate that the K^+^ ion can increase the average potential during electrochemical reactions, relative to those of Li^+^ and Na^+^ ions. In addition to the effects of atomic weight and size, the solvent can also influence the performance of KIBs [28].

In this present study, we examined maleamic acid (MA)—a low-molecular-weight organic compound containing C=O units in the form of carboxylate and amide groups as redox centers for energy storage—as a negative electrode material for both KIBs and LIBs. We focused our attention on the insertion potentials and output voltages delivered by the presence of K^+^ and Li^+^ ions. We converted the COOH group of MA into COOK and COOLi salts through simple chemical processes. In addition to studies of the electrochemical activity of K^+^ and Li^+^ cations interacting with the maleamate anion, we also assessed the effects of these salts in the presence of potassium bis(fluorosulfonyl)imide (KFSI) and lithium bis(fluorosulfonyl)imide (LiFSI) in mixtures of ethylene carbonate (EC) and ethyl methyl carbonate (EMC) as the solvent. To the best of our knowledge, the charging/discharing of K^+^ ions in a low-molecular-weight organic maleamate in KFSI/LiFSI in EC/EMC (1:2, *v*/*v*) has not been reported previously.

## 2. Materials and Methods

### 2.1. Materials

MA (Sigma-Aldrich, Chuo-Ku, Tokyo, Japan, 99%), lithium hydroxide monohydrate (LiOH·H_2_O, Alfa Aesar, Lancashire, UK, 98%), potassium hydroxide (KOH, Showa Chemical Industry Co., Ltd., Minato-ku, Tokyo, Japan, 85%), N-methyl-2-pyrrolidone (NMP, Acros Organics, 99%), Super P (SP, Sigma-Aldrich, St. Louis, MO, USA, 99%), poly (vinylidene fluoride) (PVDF, Alfa Aesar, 99%), and EtOH (Across organic, Loughborough, UK, 99.8%) were used as received. KFSI (95%) and LiFSI (98.0%) were purchased from Tokyo Chemical Industry (TCI, Tokyo, Japan).

### 2.2. Material Synthesis

**Scheme 1:**
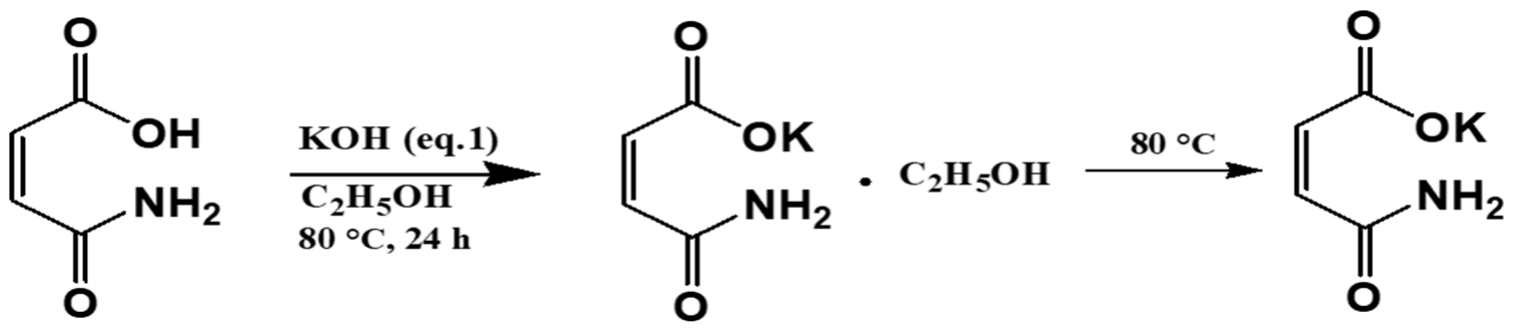
Synthesis of K-MA.

Lithium maleamate (Li-MA) and potassium maleamate (K-MA) were synthesized by dissolving MA with LiOH·H_2_O and KOH, in ethanol respectively, at a 1:1 mole ratio. The mixture was stirred for 24 h at 80 °C. The solvent was evaporated using a rotary evaporator. The white powder was washed with EtOH and dried in a vacuum oven at 80 °C for 12 h. Scheme 1 displays the proposed potassium and lithium storage mechanisms for the K-MA anode material.

### 2.3. Materials Characterization

The FTIR spectra of Li-MA and K-MA were obtained using an optics spectrometer (FT/IR-6700, JASCO, Osaka, Japan) in the frequency range 4000–400 cm^−1^. Differential scanning calorimetry (DSC, PerkinElmer DSC-4000 analyzer, Akron, OH, USA) was used to measure the thermal behavior of MA, Li-MA, and K-MA under N_2_ gas (flow rate: 50 mL min^−1^) at a heating rate of 0.5 °C min^−1^ in the temperature range from 30 to 400 °C. X-ray diffraction (XRD) was performed using a D_2_ PHASER (Bruker, Karlsruhe, Germany) with Cu Kα radiation (λ = 1.5406 Å) at an energy of 8.04 keV with a uniform increment size (2θ) of 5° at a scan rate of 5° min^−1^. The morphology of the electrodes was observed through field-emission gun scanning electron microscopy (SEM; JEOL JSM-6500, JEOL. Ltd., Tokyo, Japan) performed at an accelerating voltage of 15 keV.

### 2.4. Conductivity Tests

A mixture of EC (98%) and EMC (98%) solvents was first prepared at a volume ratio of 1:2. 1 M KFSI and 1 M LiFSI electrolytes were then prepared by adding weighed amount of these salts in the EC/EMC solvent mixture (1:2). The bottles were shaken thoroughly until the salts had dissolved completely. The prepared electrolytes were used to measure the ionic conductivities and electrochemical performance of both LIBs and KIBs. The ionic conductivities of the electrolyte solutions, 1 M KFSI and 1 M LiFSI in EC/EMC (1:2), were measured using CS300 type of conductivity sensor instrument in glove box. After cleaning conductivity cell using distilled water put deep into 0.1 M KCl and calibrated to 1413 μs/cm at 25 °C. Subsequently, ion conductivity was obtained after inserting the electrode cell into electrolyte at 25 °C.

### 2.5. Electrode Preparation and Battery Tests

Electrochemical characterization was performed by constructing a coin-type CR2032 half-cell in a glove box. The slurry for the working electrode, comprising a 60:30:10 mixture of the active material (K-MA), carbon black (SP), and PVDF, was coated on a cleaned Cu foil. The sample was dried in a vacuum oven for 12 h at 80 °C, then pressed and cut into a circular form having an area of 1 cm^2^. The solutions of 1 M KFSI and 1 M LiFSI in EC/EMC (1:2, *v*/*v*) were used as the electrolytes for the KIBs and LIBs, respectively; K and Li metal foils were used as the reference electrodes, respectively. Galvanostatic static experiments were performed using a BAT-750B battery automatic tester at a rate of 0.1C in the potential window of 0–3 V. Cyclic voltammetry (CV) was performed using a VMP3 apparatus in the potential range from 0 to 3 V at a scanning rate of 0.1 mV s^−1^ at 24 °C. EIS was performed using a VMP3 EIS instrument over the frequency range from 1 MHz to 10 mHz and an amplitude of 10 mV. The K-MA (working electrode) and potassium or lithium foils (reference electrode) are separated by Celgard 2325 type of separators and filled with electrolyte. In order to demonstrate the difference between observed and expected values of electrolyte resistance (R_e_), interface resistance (R_SEI_) and charge transferee resistance (R_ct_) in the semicircle of EIS spectra EC-lab electrochemical softer ware was used for fitting.

## 3. Results and Discussion

**Figure 1:**
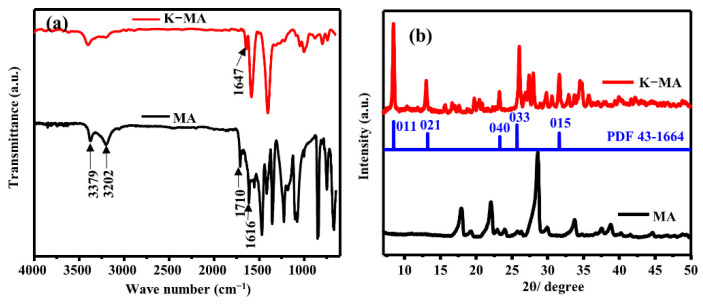
(**a**) FTIR spectra (**b**) XRD patterns of MA and K−MA.

FTIR spectra and XRD and DSC measurements were recorded to confirm the structures of MA and its salts K-MA and Li-MA form. Figure 1a presents the FTIR spectra of MA and K-MA, revealing the complete conversion of MA into K-MA. The peaks at 3379 and 3202 cm^−1^ in the spectrum of MA represent stretching of the carboxyl O–H unit and the primary amino N–H unit [29]. The signals for the C=O unit of the carboxyl group and the C=C bond appeared at vibrational frequencies of 1710 and 1580 cm^−1^ respectively. After neutralization of MA with KOH, the signal for the carboxyl C=O unit shifted to 1647 cm^−1^, consistent with the formation of the salt K-MA. Figure 1b displays the XRD patterns of MA and K-MA. Even many diffraction peaks appeared in the pattern for MA, the main peaks at 19.29, 21.06, 24.39, and 28.6° revealed its crystalline nature. After modification to K-MA the XRD pattern provided by (PDF 43-1664) shows monoclinic crystalline structure. The K-MA number of diffraction peaks increased having main peaks at 8.47, 13.23, 23.26, 25.59 and 31.62° with corresponding indexing at (011), (021), (040), (033) and (015), respectively.

Figure S1 provides the DSC curves of MA and K-MA. The trace for K-MA featured endothermic and reaction exothermic peaks in the ranges 180–196 and 196–248 °C, respectively. The broad exothermic peak implies a higher heat of reaction. The thermal decomposition of this material occurred at approximately 360 °C, higher than that of its precursor MA owing 293 °C decomposition temperature.

**Figure 2:**
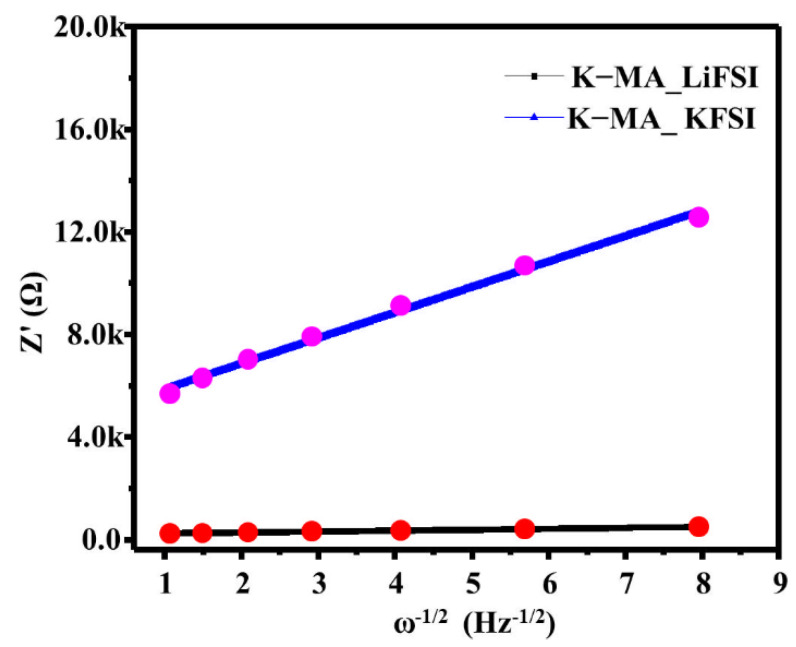
Ion diffusion coefficients of K−MA_KFSI and K−MA_LiFSI in EC/EMC (1:2) as solvent, measured from 1 MHz to 10 mHz, after 200 cycles.

Figure 2 presents Nyquist plots used to derive the ionic conductivities of 1 M KFSI and 1 M LiFSI in EC/EMC (1:2, *v*/*v*). The ion conductivities of 1 M LiPF_6_, 1 M LiFSI and 1 M KFSI determined using conductometry were 3.876, 3.65 and 8.47 mS cm^−1^, respectively (Table S1). The ion conductivities of 1 M KFSI and LiPF_6_ in dimethyl ether (DME) have been reported to be 7.2 and 5.8 mS cm^−1^, respectively [30]. Thus, the ion conductivities obtained using conductometry indicate that the 1 M KFSI ion conductivity larger than 1 M LiPF_6_ and 1 M LiFSI. The larger size of the K^+^ ion relative to the Li^+^ ion—and, therefore, the lower surface charge density (i.e., lower Lewis acidity) of the former—results in a weaker interaction between the K^+^ ion and the solvent [31]. Thus, the less-solvated K^+^ ion (4.12 eV) in the electrolyte will undergo diffusion faster than that of the more-solvated Li^+^ ion (5.85 eV) [32]. The larger radius of the K^+^ ion might, however, result in poorer ion diffusion in the electrode material, thereby limiting the performance of KIBs [33]. As indicated in Table 1, the ionic diffusion of Li^+^ ions in the solid material (2.757 × 10^−13^ cm^2^ s^−1^) was greater than that of K^+^ ions (3.927 × 10^−16^ cm^2^ s^−1^). This behavior resulted from the larger weight (ca. 5.7-fold) of the K^+^ ion, relative to the Li^+^ ion, increasing its retarding force. The larger size of the K^+^ ion makes its rates of diffusion into and out of the material host slower than those of the Li^+^ ion [11]. Moreover, the higher polarization resulting from the interaction of K^+^ ions with oxygen anions (O^−^) might also have slowed down the diffusion of K^+^ ions in the materials.

**Figure 3:**
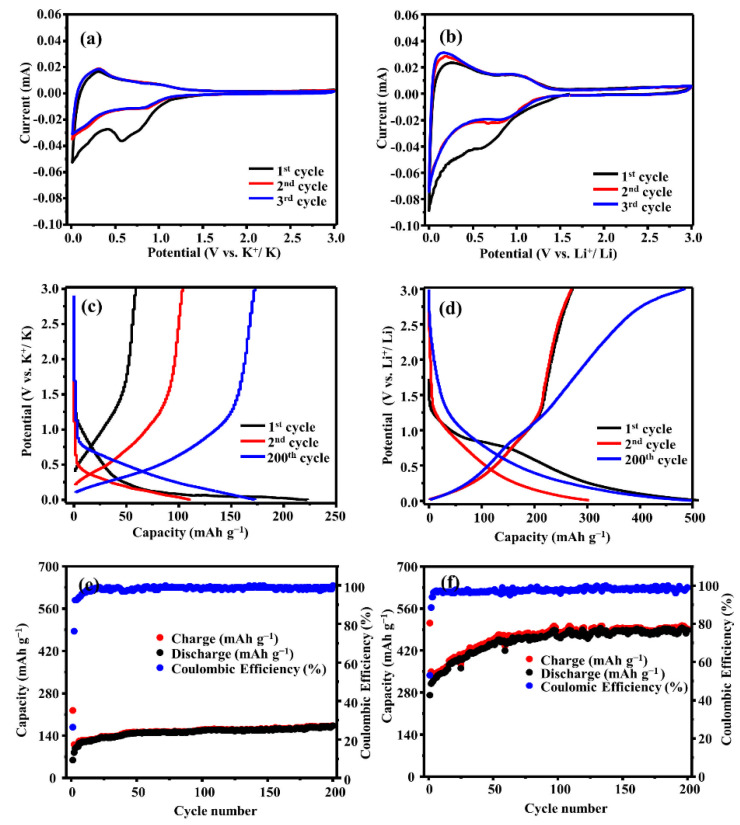
(**a**,**b**) CV curves, (**c**,**d**) galvanostatic charge/discharge curves, and (**e**,**f**) cycling behavior (**a**,**c**,**e**) K−MA_KFSI and (**b**,**d**,**f**) K−MA_LiFSI.

Figure 3a,b present the CV curves of the K-MA_KFSI and K-MA_LiFSI anode materials, respectively, recorded in the range from 0 to 3 V at a scan rate of 0.1 mV s^−1^; Figure 3c,d display their respective galvanostatic charge/discharge curves and Figure 3e,f shows their cycling behavior. In Figure 3a, the broad reduction peak at 0.57 V (detected in the plateau between 1.25 and 0.37 V) in the first cathodic scan represents the formation of the solid electrolyte interphase (SEI) and the interaction of K^+^ ions. In the subsequent cycle, this peak disappeared, and a pair of revisable peaks appeared at 0.8 and 1 V, attributable to the charging and discharging, respectively, of K^+^ ions at the C=O groups. The CV curve of K-MA_LiFSI (Figure 3b) featured a reduction peak at 0.66 V (i.e., in the plateau between 0.65 and 1 V) corresponding to SEI formation and the interaction of Li^+^ ions. In the following cycle, a pair of redox peaks appeared at 0.88 and 1.1 V, representing the reversible reactions of Li^+^ ions with the C=O groups of K-MA. After the first cycle, the reversible redox peaks for both K-MA_KFSI and K-MA_LiFSI confirmed their good reversibility.

Desai et al. reported that the energy density of an organic anode can be increased by operating it at a lower voltage [24]. Al though K-MA_KFSI began its charging process near 0.8 V in the first step of the galvanostatic charge/discharge process, in the next consecutive cycles it exhibited a very low operating voltage (<0.4 V), as illustrated in Figure 3c. This operating voltage is remarkably lower than those of other previously reported K-anode materials. In contrast, Figure 3d reveals that the K-MA_LiFSI charging began at a voltage of greater than 1 V and provided a lower output voltage when operating at a high working voltage (i.e., 0.7 V). More importantly, the charging/discharging of K^+^ ions with the active sites of K-MA_KFSI provided a significantly greater effective output voltage than those of Li^+^ ions with K-MA_LiFSI. The voltage difference arose from the larger size of the K^+^ ion relative to the Li^+^ ion. Furthermore, Lu et al. reported that the CONH_2_ group can assist to decrease the voltage plateau [34]. The ready acceptability of K^+^ ions by K-MA_KFSI at a lower potential, by delivering a higher output voltage, would further improve the energy density of corresponding KIBs. An anode material operating at lower voltage would, however, experience a low coulombic efficiency in the first cycle, due to development of the SEI layer [24]. The high reactivity of K atoms with carbonate electrolytes can lead to the development of unstable SEI layers and lower electronic conductivities [35]. Accordingly, the initial reversible capacity of the K-MA_KFSI half-cell was 59 mA h g^−1^, with a low coulombic efficiency (27%, Figure 3e) that was half of that of the K-MA_LiFSI cell (54%, Figure 3f). Herein, the high reactivity of K atoms with the aprotic organic solvents EC and EMC led to the development of an unstable SEI layer over the surface of the K-MA_KFSI electrode; as a result, a large percentage of the charged large K^+^ ions were irreversibly discharged from the material structure. Nevertheless, after 200 cycles, the sample provided a significant capacity of 172 mA h g^−1^ with a coulombic efficiency of 100%. Figure S2a,b reveal that the use of SP (100%) in KIBs and LIBs resulted in reversible capacities of 105 mA h g^−1^ over 50 cycles and 222 mA h g^−1^ over 60 cycles, respectively. Therefore, the actual capacities of K-MA_KFSI and K-MA_LiFSI were 119.5 and 374 mA h g^−1^, respectively. Herein, the actual capacity of active material was calculated using C_actual capacity_ = (0.6 × C_total_ − 0.3 × C_SP_)/0.6 [36]. Where C_actual capacity_ is the actual capacity, C_total_ is the total specific capacity of the cell, C_SP_ is the capacity of super p (SP), the percentage composition active material (K-MA) and SP are 0.6 and 0.3 respectively. K-MA has a theoretical capacity of 350 mA h g^−1^. The number of electron participated in the redox reaction was calculated using a relation of C_actual capacity_ = nF/3600M_w_, where n is the number of electrons which participated in the reaction, F is Faradays constant, and M_w_ is molecular weight of K-MA. The ex-FTIR in (Figure S3a,b) and XPS (Figure S4c,d) indicate that 1e^-^ transferred with carbonyls of primary amine group of K-MA_KFSI and K-MA-LiFSI. However, Figure S3b explain that, due to the light weight of Li^+^, another 1e^-^ participated in reaction with the remain carbonyl group of K-MA_LiFSI.

**Scheme 2:**
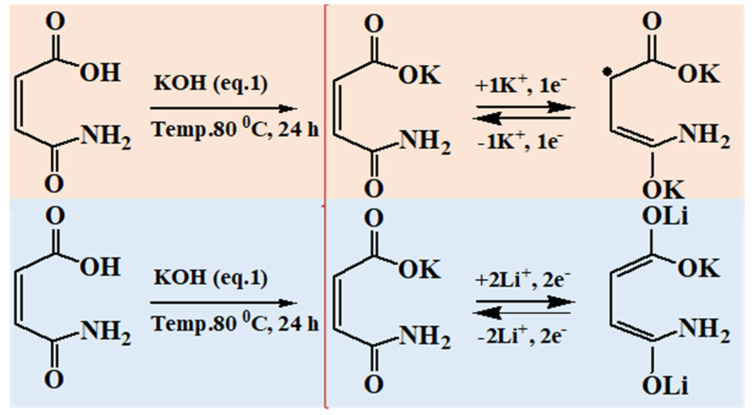
Proposed mechanisms for K^+^ and Li^+^ storage in the K−MA anode.

Based on the obtained values, the cell containing K-MA_KFSI in EC/EMC (1:2, *v*/*v*) could up take a maximum of one K^+^ ion per unit of K-MA; meanwhile, the capacity of the K-MA_LiFSI in EC/EMC (1:2, *v*/*v*) electrode was three times higher than that of the KIB, suggesting that two Li^+^ ions were engaged in the reaction, as illustrated in Scheme 2.

Figure S5a,b present the charge/discharge curves of Li-MA_KFSI anodes in KIBs and Li-MA_LiFSI in LIBs featuring in EC-EMC (1:2, *v*/*v*) as electrolytes, respectively. Figure S5c reveals that the Li-MA_KFSI anode provides a capacity of 148 mA h g^−1^ at 0.1C after 31 cycles. Similarly, the LIB featuring the Li-MA_LiFSI anode and 1 M LiFSI displayed a capacity of 375 mA h g^−1^ at 0.1C over 37 cycles (Figure S5d). Comparing the capacities of the Li-MA_KFSI (148 mA h g^−1^) and K-MA_KFSI (119.5 mA h g^−1^) anodes, the former delivered a higher reversible capacity. This difference might have arisen from the smaller size of the Li^+^ ion and the weaker repulsive O···Li^+^ force [34], relative to that of the O···K^+^ interactions in K-MA, leading to acceptance of an additional K^+^ ion by the remaining C=O group. Alternatively, the lower weight and smaller radius of the Li^+^ ion, relative to the K^+^ ion, might have enhanced its kinetic reactions. Furthermore, Zhang et al. reported that the large size of the K^+^ ion was responsible for the thick SEI layer formed on the surface of the electrode material; accordingly, the K^+^ ions created “dead” areas [3]. As a result of the formation of an SEI barrier layer on the K-MA_KFSI surface, the performance of the KIBs was limited when compared with that of the LIBs. Comparison of the performance of the K-MA_LiFSI and Li-MA_LiFSI electrodes in Figure 3f and Figure S5d reveals that the latter gave a higher storage capacity, due to the lower weight and smaller size of the Li^+^ ion (thereby providing more space for the reversible reaction) compared with the K^+^ ion.

**Figure 4:**
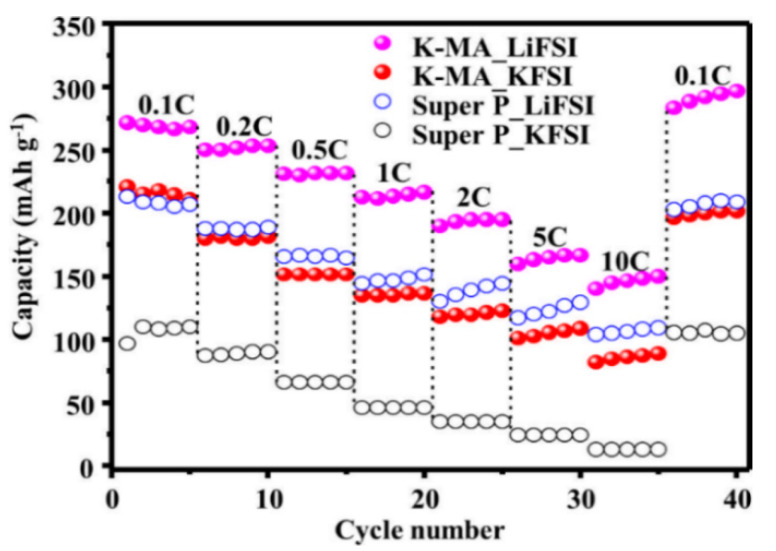
Rate capabilities of K−MA_LiFSI, K−MA_KFSI, SP_LiFSI, and SP_KFSI measured at various rates from 0.1C to10C.

Figure 4 presents the rate capabilities of the K-MA_LiFSI, K-MA_KFSI, SP_LiFSI, and SP_KFSI anodes measured at different C-rate from 0.1C to 10C. The capacities of K-MA_LiFSI, K-MA_KFSI, SP_LiFSI, and SP_KFSI at equivalent C-rate of 0.1, 0.2, 0.5, 1, 2, 5, and 10C were 268.3/210/213.1/110, 253.3/181/188.8/90.2, 231.7/151.5/164.6/66, 216.7/136.3/151.5/46, 195/122.7/144.4/35, 166.7/108.6/129.3/24, and 150/88.9/109.3/13 mA h g^−1^, respectively; the superior recoveries of electrochemical performance at 0.1C were 320/201/209/105 mA h g^−1^, with satisfactory capacity retentions of 110, 112, 98, and 95% respectively. To realize the contributions of SP_KFSI in K-MA_KFSI and SP_LiFSI in K-MA_KFSI, we considered the rate capability of SP at 10C as a reference for comparison. Accordingly, K-MA_KFSI dominated over SP_KFSI by 93.4%, revealing that K-MA_KFSI had been amended. Although the K^+^ ion has a large size, nothing stopped it from undergoing reversible cycling at high rates. The evaluated rate capability of K-MA_KFSI decreased from 108.6 to 88.9 mA h g^−1^ (i.e., it decreased by 15%) when the C-rate increased from 5C to 10C (Table S2). On the other hand, the capacity of K-MA_LiFSI decreased from 166.67 to 150 mA h g^−1^ (i.e., a decrease of 8.57%), with a decrease in the contribution of SP_LiFSI in the material from 129.3 to 109 mA h g^−1^ (i.e., a decrease of 12.84%), as the rate changed from 5C to 10C. The larger atomic sizes and masses of Na^+^ and K^+^ ions lead to lower retention capacities at high current densities, relative to those of Li^+^ ions [25]. Therefore, K-MA_LiFSI allowed faster diffusion of Li^+^ ions in the material.

**Figure 5:**
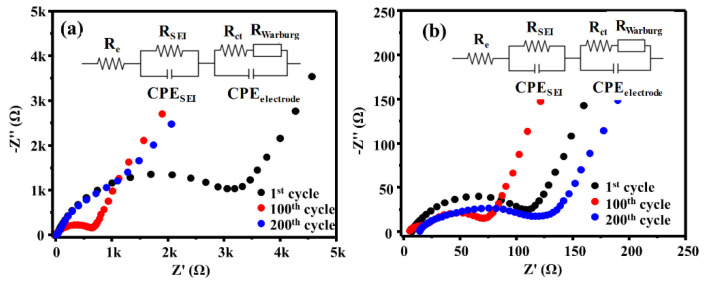
EIS plots of K−MA as (**a**) KIB and (**b**) LIB electrodes; first, 100th, and 200th cycles, measured from 1 MHz to 10 mHz.

Figure 5a,b provide EIS spectra measured after various degree of electrochemical cycling of K-MA_KFSI and K-MA_LiFSI. The semi-circles of the K-MA_KFSI and K-MA_LiFSI electrodes in the Nyquist plot decreased from the high to the low frequency region after the materials had been subjected to 100 cycles. Table 2 reveals that the K-MA_KFSI interface resistance (R_SEI_) decreased from 3250 Ω after the first cycle to 30.56 Ω after the 100th cycle; in comparison, the interface resistance of K-MA_LiFSI decreased from 119.8 Ω after the first cycle to 25.5 Ω after the 100^th^ cycle. However, the charge transfer resistance (R_ct_) of K-MA_KFSI increased from 10.27 Ω after the first cycle to 593.5 Ω after 100 cycles, whereas for K-MA_LiFSI these values were 35.53 and 50.17 Ω, respectively. These values reveal that the degrees of charge transfer and ionic diffusion had both reduced in the K-MA_KFSI and K-MA_LiFSI electrodes. Nevertheless, the charge transfer capability in K-MA_LiFSI was faster than that in K-MA_KFSI, due to the polarization effects that resulted from the different ionic sizes. During a long cycling process, the electrode-electrolyte interface results in the continuous formation of an SEI layer that can damage the integrity of the material. The EIS profiles reveal that the values of R_SEI_ and R_ct_ increased after 200 cycles for both the K-MA_LiFSI and K-MA_KFSI electrodes, due to the electrode-electrolyte interface causing more serious SEI formation for K-MA_KFSI than for K-MA_LiFSI. Warburg diffusion was presented at low frequency, suggesting that the ionic coefficient of diffusion for K-MA_LiFSI was greater than that for K-MA_KFSI, as mentioned in Table 1.

**Figure 6:**
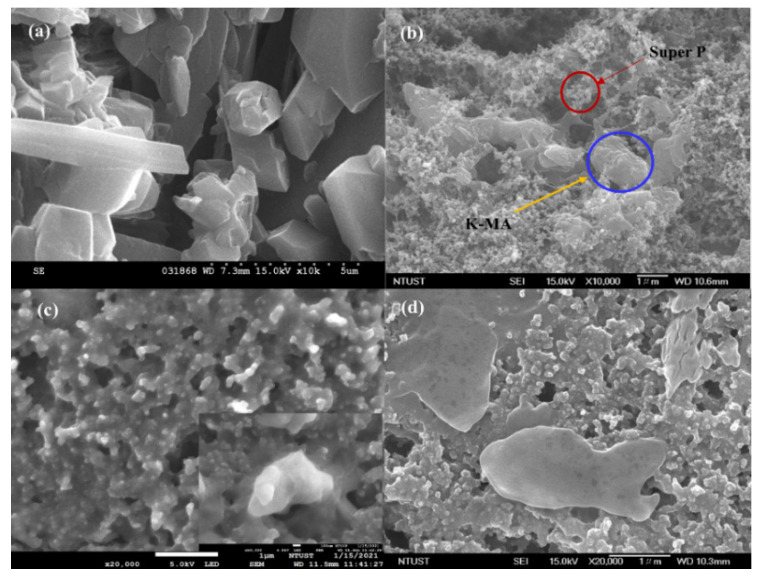
SEM images of (**a**) pure K−MA, (**b**) pristine K−MA, (**c**) K-MA_LiFSI and (**d**) K−MA_KFSI after completing 200 cycles.

We measure the morphologies of the K-MA_KFSI and K-MA_LiFSI electrodes before and after long periods of cycling, in addition the SEI image of pure K-MA was measured as shown in Figure 6a. Figure 6c reveals that the K-MA_LiFSI surface featured a uniform and thin SEI film after 200 cycles. The absence of cracks in the film suggested enhanced mechanical stability, integrity, and mechanical strength for the K-MA_LiFSI electrode. In contrast, the K-MA_KFSI surface (Figure 6d) was covered by a non-uniform, lower-integrity protective SEI layer film, with cracks and irregular SEI coverage.

**Figure 7:**
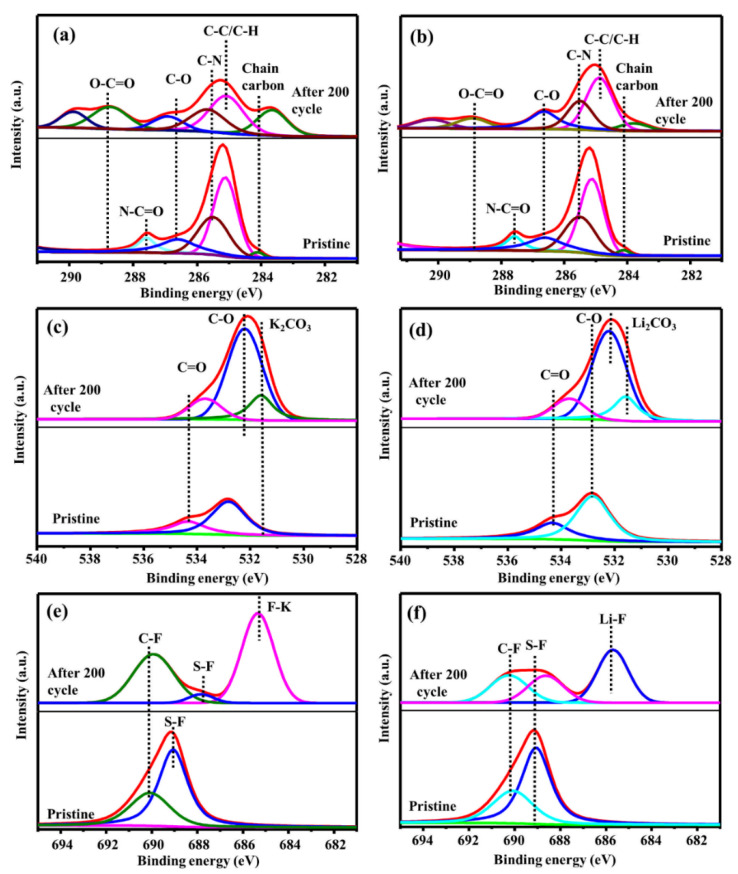
High−resolution (**a**,**b**) C 1s, (**c**,**d**) O 1s, and (**e**,**f**) F 1s XPS spectra of (**a**,**c**,**e**) K−MA_KFSI and (**b**,**d**,**f**) K−MA_LiFSI electrodes, recorded before and after 200 cycles.

We recorded XPS spectra of the surfaces of the K-MA_KFSI and K-MA_LiFSI electrodes to identify the specified elements that were present in the SEI layer. Figure 7 displays the C 1s, O 1s, and F 1s XPS spectra, and their fitting data, for the K-MA_KFSI and K-MA_LiFSI materials after 200 cycles. Figure 7a presents the C 1s spectra, obtained with higher resolution, of K-MA_KFSI. Binding energies of 285, 286.55, 288.79, and 290.1 eV correspond to C–C, C–O, C=O, and C–F bonds present in an electrode [36]. Thus, the presence of an alkyl carbonate was confirmed by the peak at 288.67 eV, with the peak at 284.1 eV representing the carbon chains formed during synthesis [37]. The high-resolution O 1s spectrum revealed the presence of C–O (532.2 eV) and C=O (533.7 eV) bonds (Figure 7c). At this resolution, we could identify an organic potassium alkyl carbonate (ROCO_2_K) [33], with a signal at a binding energy of 531.6 eV indicating the presence of K_2_CO_3_, which, in turn, specifies the formation of an inorganic-rich salt on the surface [36]. K–F (685.38 eV) and S–F (685.38 eV) bonds were identified by their respective binding energies [38] in the F 1s XPS spectrum (Figure 7e). An inorganic-rich salt (KF) was formed on the materials surface by breaking the bonds of the FSI anion. Evidence for KF formation was a decrease in the intensity of the S–F peak at 687.8 eV and an increase in the intensity of the KF peak [39]. The F 1s spectra recorded after the charging/discharging processes (Figure S4e) provide further evidence for the generation of KF from the decomposition of KFSI.

The major components of K-MA_LiFSI that appeared on the electrode surface (Figure 7b) were C–C, C–N, C=O, and N–C=O bonds, with binding energies of 285, 285.5, 286.6, and 287.6 eV, respectively. After 200 cycles, the intensity of the C–O peak decreased, relative to that of the pristine sample, indicative of Li^+^ reaction and the development of the SEI layer through decomposition of the electrolyte. Along with the development of alkyl lithium carbonates, this behavior confirmed the development of an undissolved SEI layer, with evidence for carbon chains also appearing at 284.1 eV [37].

Figure 7d reveals that signals appeared for C–O (534.21 eV) [40] and C=O (532.5 eV) bonds [41] in the O 1s spectrum recorded prior to cycling; after 200 cycles, the signal for the C–O bonds shifted slightly to lower binding energy, suggesting that a doping reaction had occurred with Li^+^ [41]. The transformation of N–C=O to N–C–OK in the charging process was evidenced by a peak at 531.7 eV peak in the O 1s spectrum in Figure S4c, revealing that K^+^ ions had been reacted with oxygen anions. A new peak at 530.8 eV after K-MA_LiFSI charging is evident in Figure S4d from fitting of the O 1s XPS spectrum. Furthermore, the F 1s XPS spectrum in Figure 7f provides evidence for the development of LiF from the reduction of LiFSI. The decrease in the intensity of the signal for the S–F bonds after 200 cycles, and the increase in that of LiF inorganic salt, confirmed the development of a LiF electrode surface that served as an electrically insulated component.

The intensity of the signals for the organic species in the C 1s spectrum of the de-doped K-MA_KFSI electrode (Figure S4b) decreased when compared with those of the K-MA_LiFSI electrode (Figure S4d), indicating that the SEI formed covering the former electrode featured unstable organic species, relative to those formed on the latter. The stronger and higher-intensity signal for residual KFSI in K-MA_KFSI (Figure S4e) on a discharged electrode surface, relative to that of K-MA_LiFSI (Figure S4f), suggests that a thicker SEI layer formed for the former than that for the latter. In addition, the intensity of the peak for LiF in the SEI was higher than that of KF, revealing that the presence of inorganic-rich LiF in the SEI layer protected the K-MA_LiFSI electrode.

Figure S3 demonstrate that the ex-situ FTIR spectra of K-MA_KFSI and K-MA_LiFSI electrodes in pristine, charging, and discharging states. In Figure S3a, the O=C−N intensity peak of the K-MA_KFSI pristine at 1562 cm^−1^ is weaken and broad when charged to 0 V and reappeared after discharged to 3 V. This indicates that carbonyl of the primary amine participated in charge-discharge reaction. However, in Figure S3b the K-MA_LiFSI pristine electrode of intensity peaks C=O at 1676 cm^−1^ and O=C−N at 1562 cm^−1^ become diminished after charged to 0 V and recover when discharged 3 V, indicating both carbonyls group participated in redox reaction.

## 4. Conclusions

We synthesized K−MA from MA and investigated its use as an anode material for electrochemical storage in KIBs and LIBs, with 1 M KFSI and 1 M LiFSI in common mixed EC/EMC solvents as respective electrolytes. Charge/discharge and CV profiles revealed that the K^+^ ions were operated at a voltage lower than that of the Li^+^ ions. Because the size of a K^+^ ion is larger than that of a Li^+^ ion, the interacting bond energy of K–O is weaker than that of Li–O, potentially giving a output voltage for K^+^ higher than that for Li^+^. As an anode, K-MA_KFSI delivered a storage capacity of 172 mA h g^−1^ over 200 cycles at a rate of 0.1C, with a high coulombic efficiency (100%); in contrast, K-MA_LiFSI provided a satisfactory storage capacity of 485 mA h g^−1^ at a current density of 0.1C after completing 200 cycles, with a coulombic efficiency of 98.7%. K-MA was capable of accepting one K^+^ ion in the KIB, whereas it could accept two Li^+^ ions in the LIB. Although the K-MA_LiFSI electrode provided faster Li^+^ ion diffusion, the KIBs displayed a larger output voltage. This study contributes to our understanding of the electrochemical effects of alkali metal ions (Li^+^ and K^+^) and their suitability for use in the development of organic-based KIBs. We hope that the investigation of K-MA as KIBs using more electric conductivity additive (MCMB) will be carried out for future.

## Figures and Tables

**Table 1:** K^+^ and Li^+^ ion conductivities in 1 M KFSI and 1 M LiFSI electrolytes, and K^+^ and Li^+^ ion diffusion coefficients in K-MA electrode from Figure 2.

Electrolyte Salt Mixed in EC/EMC (1:2, *v*/*v*)	Ion Conductivity (mS cm^−1^)	Ion in the Electrode	Ion Diffusion Coefficient in Electrode (cm^2^ s^−1^)
1 M LiFSI	3.65	Li^+^	2.76 × 10^−13^
1 M KFSI	8.47	K^+^	3.93 × 10^−16^

**Table 2:** Resistances determined from EIS spectra of electrodes after first, 100th, and 200th cycles.

K-MA_KFSI				K-MA_LiFSI		
Cycle No.	*R*_e_ (Ω)	*R*_SEI_ (Ω)	*R*_ct_ (Ω)	*R*_e_ (Ω)	*R*_SEI_ (Ω)	*R*_ct_ (Ω)
1	12.6	3250	10.3	7.0	119.8	35.5
100	4.7	30.56	593.5	4.1	25.5	50.17
200	5.7	1056	839.7	12.0	47.11	74.89

## Data Availability

No data analyzed or generated during this study. Data sharing is not applicable to this article.

## Supplementary Materials

The following are available online at https://www.mdpi.com/article/10.3390/nano11113120/s1, Figure S1: DSC of MA and K-MA, recorded at a scan rate of 10 °C min^−1^, Figure S2: Charge/discharge profiles and cyclic performances of SP_KFSI (100%) as KIBs and SP_LiFSI (100%) as LIBs respectively, at 0.1C rate, Figure S3: Ex-situ FTIR spectra of (a) K-MA_KFSI and (b) K-MA_LiFSI electrodes in pristine, charging state (CS), and discharging states (DCS), Figure S4: The C 1s, O 1s, F 1s spectra for K-K-MA_KFSI and K-MA_LiFSI electrodes in pristine, doping, and de-doping states, Figure S5: Charge/discharge curves and cyclic performances for Li-MA_KFSI and Li-MA_LiFSI, Table S1: Li^+^ ion conductivities in 1 M LiPF_6_ and 1 M LiFSI and K^+^ ion conductivity in 1 M KFSI, measured through conductometry, Table S2: Capacities of K-MA_LiFSI, K-MA_KFSI, SP_LiFSI, and SP_KFSI measured at current densities of 5C and 10C.
