# Supplementary material for: Lithium and Potassium Cations Affect the Performance of Maleamate-Based Organic Anode Materials for Potassium- and Lithium-Ion Batteries
Source: Nanomaterials (Basel). 2021 Nov 19;11(11):3120. doi: 10.3390/nano11113120 (PMC8623018; doi:10.3390/nano11113120)
Supplement: Supplementary file 1 [file nanomaterials-11-03120-s001.zip › nanomaterials-1398329-supplementary.pdf]

Lithium and Potassium Cations Affect the Performance of Maleamate-Based Organic Anode Materials for Potassium- and Lithium-Ion Batteries

Kefyalew Wagari Guji [1,2], Wen-Chen Chien [2,3,*], Fu-Ming Wang [1,4,5,6,*], Alagar Ramar [1], Endazenaw Bizuneh Chemere [1,2], Lester Tiong [1] and Laurien Merinda [1]

[1] Graduate Institute of Applied Science and Technology, National Taiwan University of Science and Technology, Taipei 106, Taiwan; gujikefyalew@gmail.com (K.W.G.); chemramar@gmail.com (A.R.); endazenaw27@gmail.com (E.B.C.); ltpw93@hotmail.com (L.T.); merinda795@gmail.com (L.M.)

[2] Battery Research Center of Green Energy, Ming Chi University of Technology, New Taipei City 243, Taiwan

[3] Department of Chemical Engineering, Ming Chi University of Technology, New Taipei City 243, Taiwan

[4] Sustainable Energy Center, National Taiwan University of Science and Technology, Taipei 106, Taiwan

[5] Department of Chemical Engineering, Chung Yuan Christian University, Taoyuan 320314, Taiwan

[6] R&D Center for Membrane Technology, Chung Yuan Christian University, Taoyuan 32023, Taiwan

* Correspondence: wcchien@mail.mcut.edu.tw (W.-C.C.); mccabe@mail.ntust.edu.tw (F.-M.W.); Tel.: +886-2-2908-9899 (ext. 4628) (W.-C. C.); +886-2-2730-3755 (F.-M. W.); Fax: +886-2-2908-3072 (W.-C. C.); +886-2-2737-6922 (F.-M. W.)

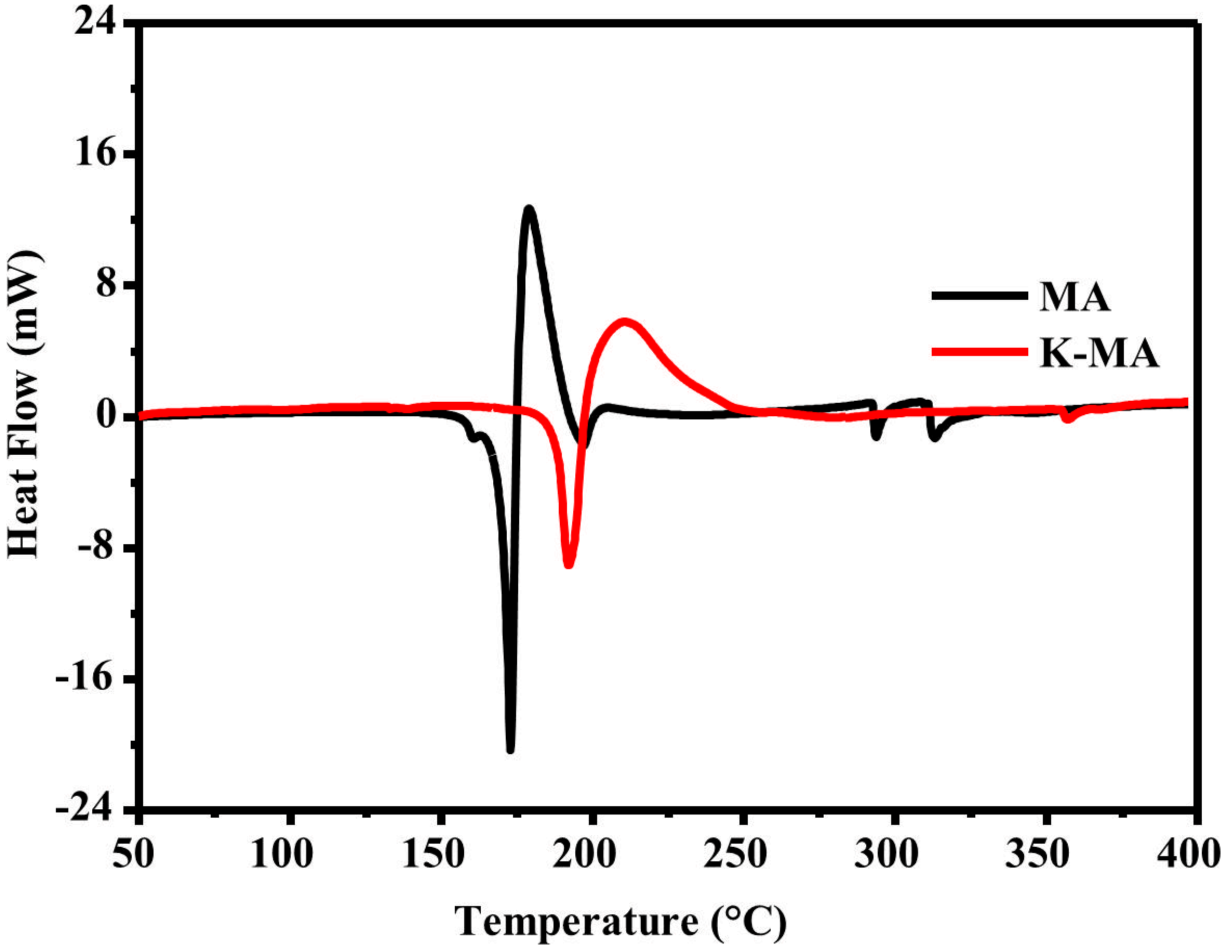

Figure S1. DSC traces of MA and K-MA, recorded at a scan rate of 10 °C min^{-1}.

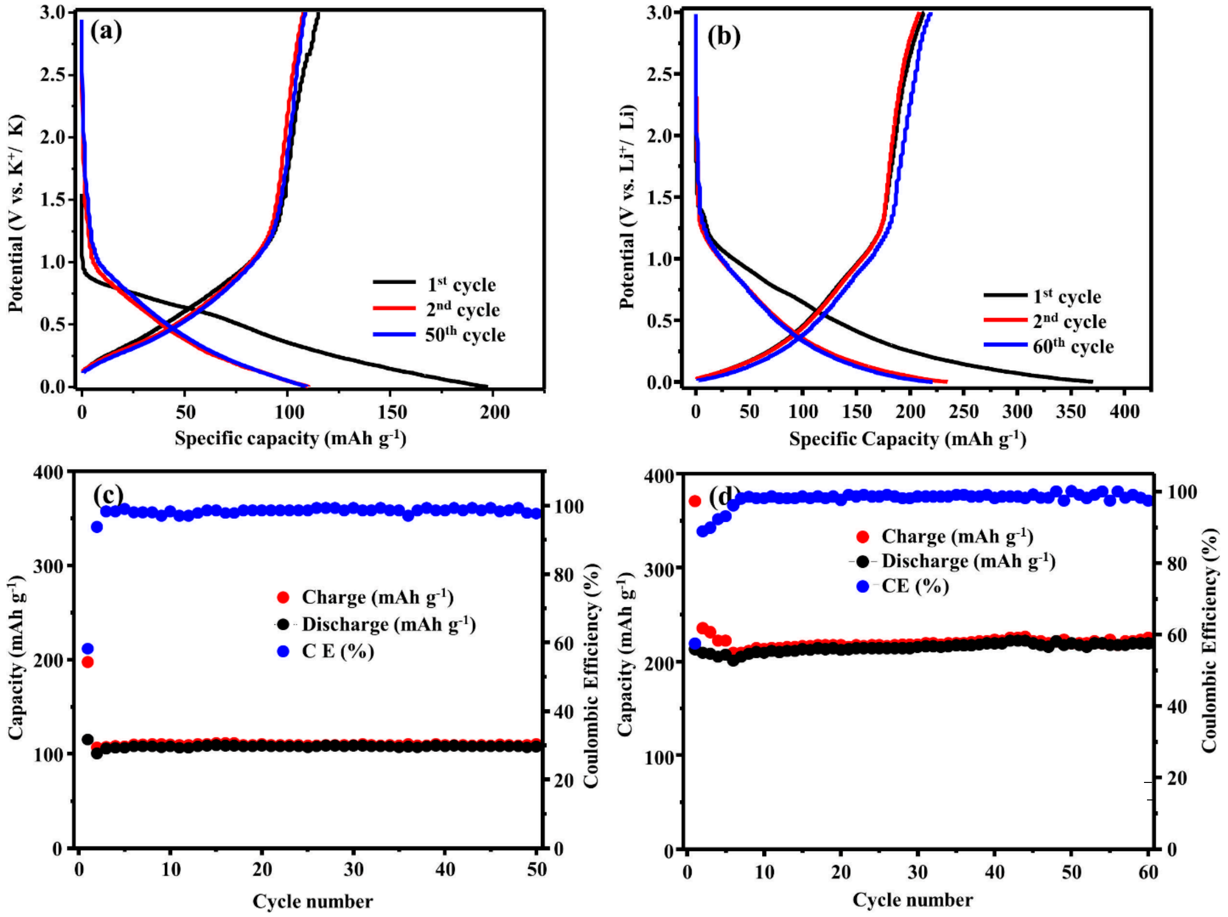

Figure S2. (**a, b**) Charge/discharge profiles and (**c, d**) cyclic performance of (**a, c**) SP_KFSI (100%) as KIBs and (**b, d**) SP_LiFSI (100%) as LIBs, measured at a scan rate of 0.1 mV s^{-1} and a rate of 0.1C.

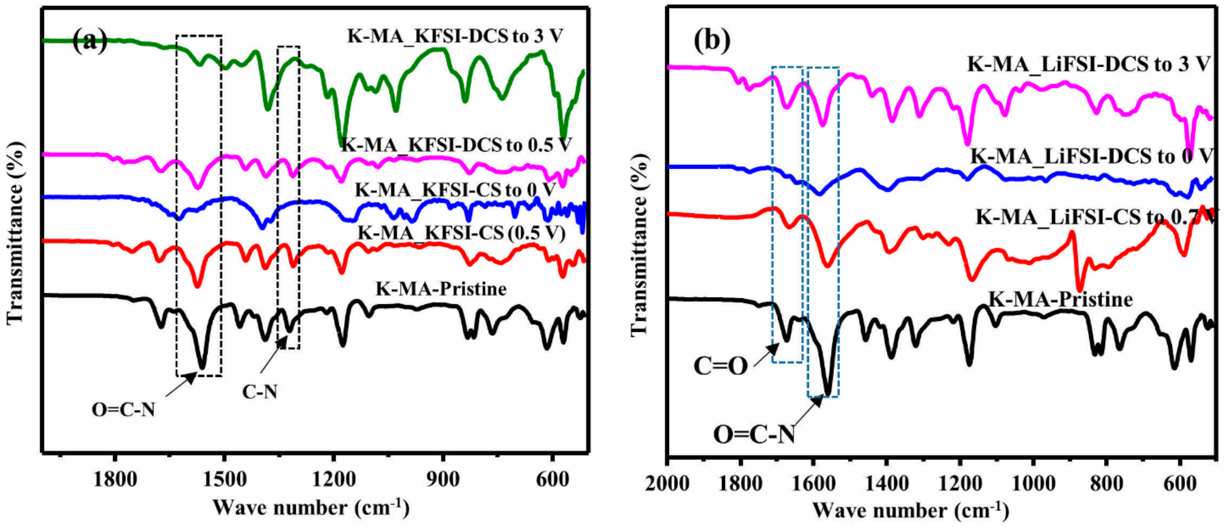

Figure S3. Ex-situ FTIR spectra of (**a**) K-MA_KFSI and (**b**) K-MA_LiFSI electrodes in pristine, charging state (CS), and discharging states (DCS).

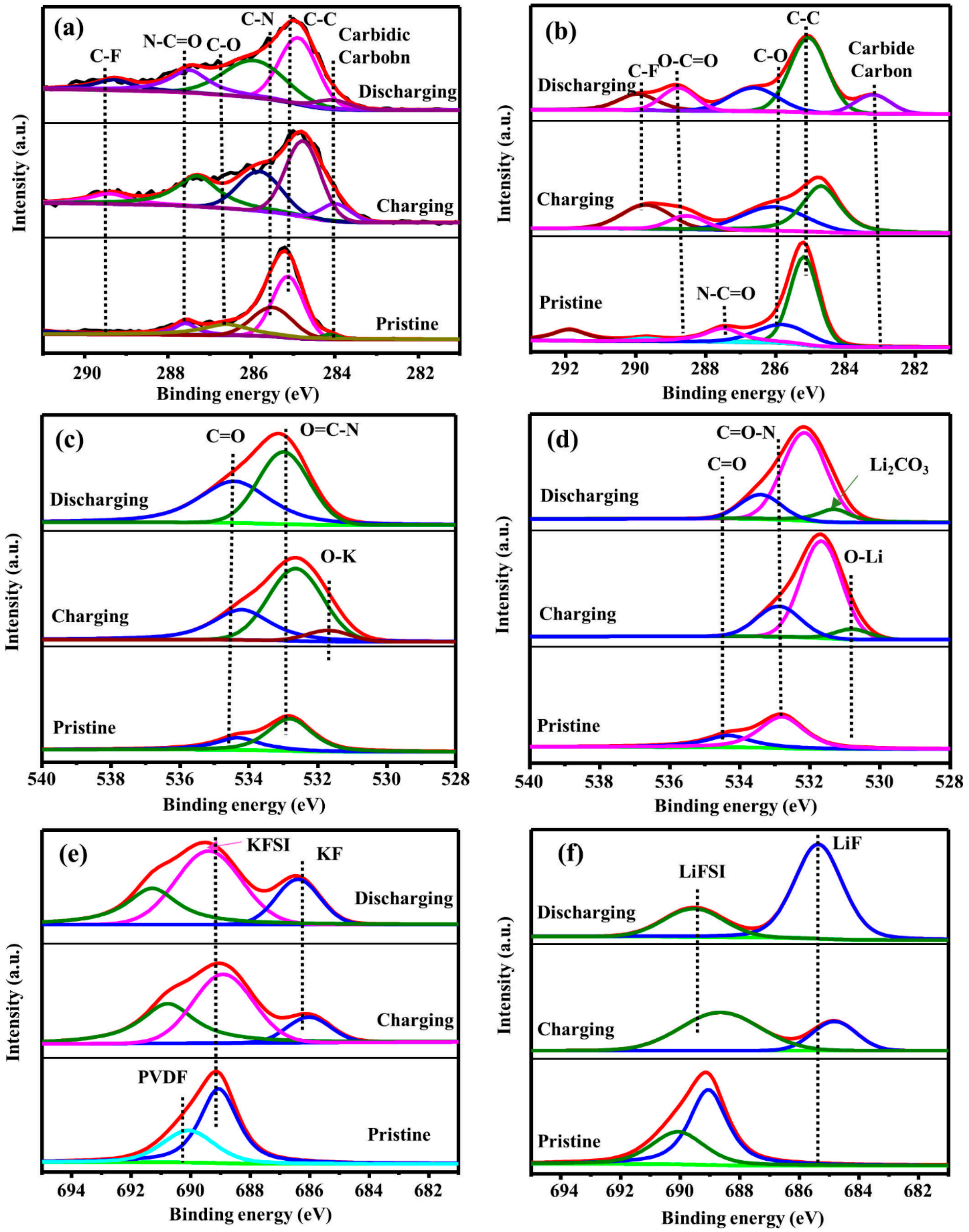

Figure S4. (**a, b**) C 1s, (**c, d**) O 1s, and (**e, f**) F 1s XPS spectra of (**a, c, e**) K-MA_KFSI and (**b, d, f**) K-MA_LiFSI electrodes in pristine, charging, and discharging states.

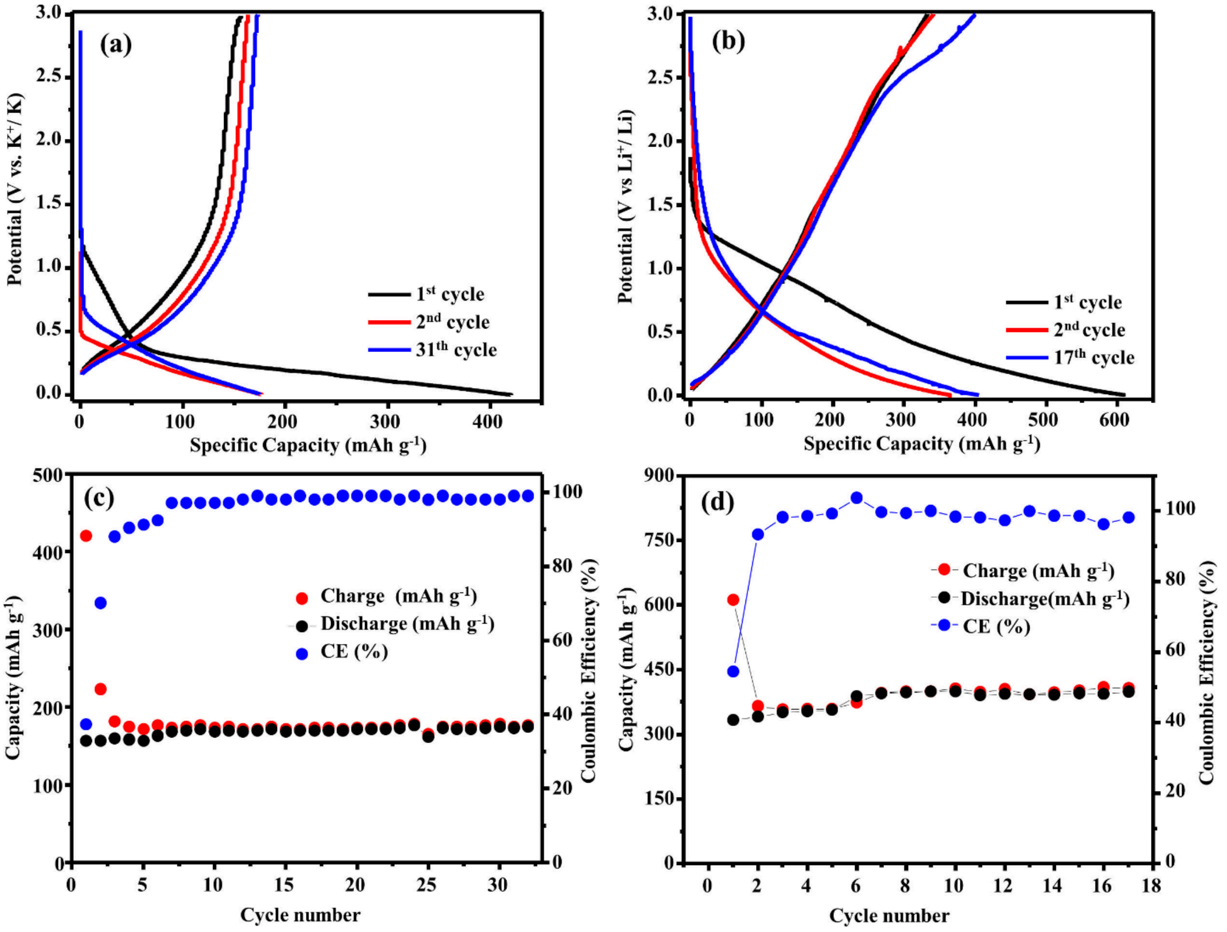

Figure S5. (**a, b**) Charge/discharge profiles and (**c, d**) cyclic performance of (**a, c**) Li-MA_KFSI and (**b, d**) Li-MA_LiFSI at a rate of 0.1C.

Table S1. Li^+ ion conductivities in 1 M $LiPF_6$ and 1 M LiFSI and K^+ ion conductivity in 1 M KFSI, measured through conductometry.

Electrolyte	Ionic conductivity (mS cm^{-1})	Temperature (°C)
1M $LiPF_6$ [EC:PC:DEC, 3:2:5 (v/v)]	3.88	24.7
1M LiFSI [EC:EMC, 1:2 (v/v)]	3.65	25.5
1M KFSI [EC:EMC, 1:2 (v/v)]	8.47	25.2

Table S2. Capacities of K-MA_LiFSI, K-MA_KFSI, SP_LiFSI, and SP_KFSI measured at current densities of 5C and 10C.

**Sample**	**5C (mA h g^{-1})**	**10C (mA h g^{-1})**	**Difference** (mA h g^{-1})
K-MA_LiFSI	166.7	150.0	–5.67 (–8.57%)
K-MA_KFSI	108.6	88.9	–8.8 (–15%)
SP_LiFSI	125.4	109.3	–14.49 (–12.84%)
SP_KFSI	24.3	13.1	–10.08 (–46.1%)
